# Trajectories of fatigue among stroke patients from the acute phase to 18 months post-injury: A latent class analysis

**DOI:** 10.1371/journal.pone.0231709

**Published:** 2020-04-15

**Authors:** Anita Kjeverud, Kristin Østlie, Anne-Kristine Schanke, Caryl Gay, Magne Thoresen, Anners Lerdal

**Affiliations:** 1 Department of Physical Medicine and Rehabilitation, Innlandet Hospital Trust, Ottestad, Norway; 2 Department of Psychology, University of Oslo, Oslo, Norway; 3 Department of Research, Sunnaas Rehabilitation Hospital, Nesodden, Norway; 4 Department of Research, Lovisenberg Diaconal Hospital, Oslo, Norway; 5 Department of Family Health Care Nursing, University of California, San Francisco, California, United States of America; 6 Department of Biostatistics, Oslo Centre for Biostatistics and Epidemiology, University of Oslo, Oslo, Norway; 7 Department of Interdisciplinary Health Sciences, Institute of Health and Society, Faculty of Medicine, University of Oslo, Oslo, Norway; University of Kansas Medical Center, UNITED STATES

## Abstract

**Introduction:**

Post-stroke fatigue (PSF) is a common symptom affecting 23–75% of stroke survivors. It is associated with increased risk of institutionalization and death, and it is of many patients considered among the worst symptoms to cope with after stroke. Longitudinal studies focusing on trajectories of fatigue may contribute to understanding patients’ experience of fatigue over time and its associated factors, yet only a few have been conducted to date.

**Objectives:**

To explore whether subgroups of stroke survivors with distinct trajectories of fatigue in the first 18 months post stroke could be identified and whether these subgroups differ regarding sociodemographic, medical and/or symptom-related characteristics.

**Materials and methods:**

115 patients with first-ever stroke admitted to Oslo University Hospital or Buskerud Hospital were recruited and data was collected prospectively during the acute phase and at 6, 12 and 18 months post stroke. Data on fatigue (both pre- and post-stroke), sociodemographic, medical and symptom-related characteristics were collected through structured interviews, standardized questionnaires and from the patients’ medical records.

Growth mixture modeling (GMM) was used to identify latent classes, i.e., subgroups of patients, based on their Fatigue Severity Scales (FSS) scores at the four time points. Differences in sociodemographic, medical, and symptom-related characteristics between the latent classes were evaluated using univariate and multivariable ordinal regression analyses.

**Results and their significance:**

Using GMM, three latent classes of fatigue trajectories over 18 months were identified, characterized by differing levels of fatigue: low, moderate and high. The mean FSS score for each class remained relatively stable across all four time points. In the univariate analyses, age <75, pre-stroke fatigue, multiple comorbidities, current depression, disturbed sleep and some ADL impairment were associated with higher fatigue trajectories. In the multivariable analyses, pre-stroke fatigue (OR 4.92, 95% CI 1.84–13.2), multiple comorbidities (OR 4,52,95% CI 1.85–11.1) and not working (OR 4.61, 95% CI 1.36–15,7) were the strongest predictor of higher fatigue trajectories The findings of this study may be helpful for clinicians in identifying patients at risk of developing chronic fatigue after stroke.

## Introduction

Post-stroke fatigue (PSF) is a common symptom affecting 23–75% of stroke survivors [1]. For many stroke survivors, it is considered among the worst symptoms to cope with following a stroke [2]. Fatigue has been described as a state of weariness unrelated to previous exertion levels, which is usually not ameliorated by rest, and as a chronic and subjective feeling of lack of energy, weariness and aversion to effort [3]. PSF is associated with higher risk of institutionalization and death and impedes patients’ rehabilitation [4] and quality of life [5]. Although fatigue in the acute phase is often considered a normal and temporary feature following stroke, Lerdal et al. [6] identified fatigue in the acute phase as an independent predictor of poorer physical health 36 months after stroke. Increased knowledge about the onset and different trajectories of PSF and which factors predict these trajectories may enable us to develop empirically-based interventions that ameliorate PSF and its debilitating sequelae.

Longitudinal studies shed light on trajectories of fatigue and are critical to understanding patients’ experience of fatigue over time and its associated factors. Longitudinal studies have the advantage of reporting individual changes over time, in addition to the proportion of patients who have the symptom at particular time points obtainable in cross-sectional studies. A systematic review of longitudinal studies on PSF by Duncan et al. [7] found only nine that assessed fatigue at multiple time points after stroke. Fatigue declined across time points in seven of the studies and increased in two studies. Of these studies, five assessed fatigue at two time points, three at three time points and one at four separate time points, and only two of the studies assessed fatigue within the two first weeks after stroke. More recently, Duncan et al. [8] conducted a longitudinal study, which assessed fatigue within the first month and at 6 and 12 months after stroke and found that a low level of physical activity at 6 and 12 months was associated with a higher degree of fatigue.

From a clinical point of view, it is important to determine whether fatigue in the acute phase following stroke can predict chronic PSF, and if so, what clinical characteristics are associated with the different fatigue trajectories. This is a critical step to the early identification of patients vulnerable to developing PSF and the development of more tailored treatment programs like those in use for patients with cognitive and physical deficits. Findings related to early mobilization indicate that the acute phase following stroke may be a critical period for maximizing recovery [9]. There are, however, few studies that explore fatigue in the acute phase after stroke and whether it is related to chronic fatigue problems. A cross-sectional study by Mutai et al. [10] reported the prevalence of PSF within 2 weeks post-stroke to be 56.4% using the Multidimensional Fatigue Inventory, and multivariable stepwise regression analyses showed that anxiety, right hemisphere lesion sites and thalamus lesions were associated with general fatigue. The study did not, however, include a follow-up.

PSF is considered a complex symptom influenced by different factors and the interactions between them [11]. Wu et al. has proposed a model for understanding PSF, which states that it is plausible that different factors contribute to PSF at different times. Three longitudinal studies found significant associations between fatigue and mood at the same time point [7]. PSF has been associated with depressive symptoms even among patients who do not meet diagnostic criteria for depression [12]. While early fatigue might be associated with neuroendocrine systems and damage to brain structures responsible for maintaining wakefulness and attention, psychosocial and behavioral factors might be important for both triggering and maintaining fatigue. Vulnerability to stress might be a predisposing factor and lead to both pre- and post-stroke fatigue, and psychological factors such as self-efficacy beliefs and coping style might influence the course of fatigue [11]. In accordance with this, retrospective self-reports of fatigue lasting longer than 3 months prior to stroke were found to be associated with PSF in a group of 115 patients [6]. Choi-Kwon et al. found that pre-stroke fatigue was the strongest predictor of PSF an average of 15 months after stroke in a sample of 220 outpatients [13]. Stroke patients often suffer from other medical conditions, such as diabetes, high blood pressure, and heart conditions [14], and these comorbidities could also lead to both pre- and post-stroke fatigue.

All in all, there seem to be gaps in current knowledge about trajectories of fatigue following stroke, specifically those that include the acute phase, and about which factors might be predictive of PSF from an early phase.

### Aims

The aims of this study were to explore whether subgroups of stroke survivors with distinct trajectories of fatigue in the first 18 months after stroke could be identified, and if so, to determine whether these subgroups differ on sociodemographic, medical and symptom-related characteristics, such as sleep, fatigue and depression.

## Materials and methods

### Patients and procedures

**Fig 1:**
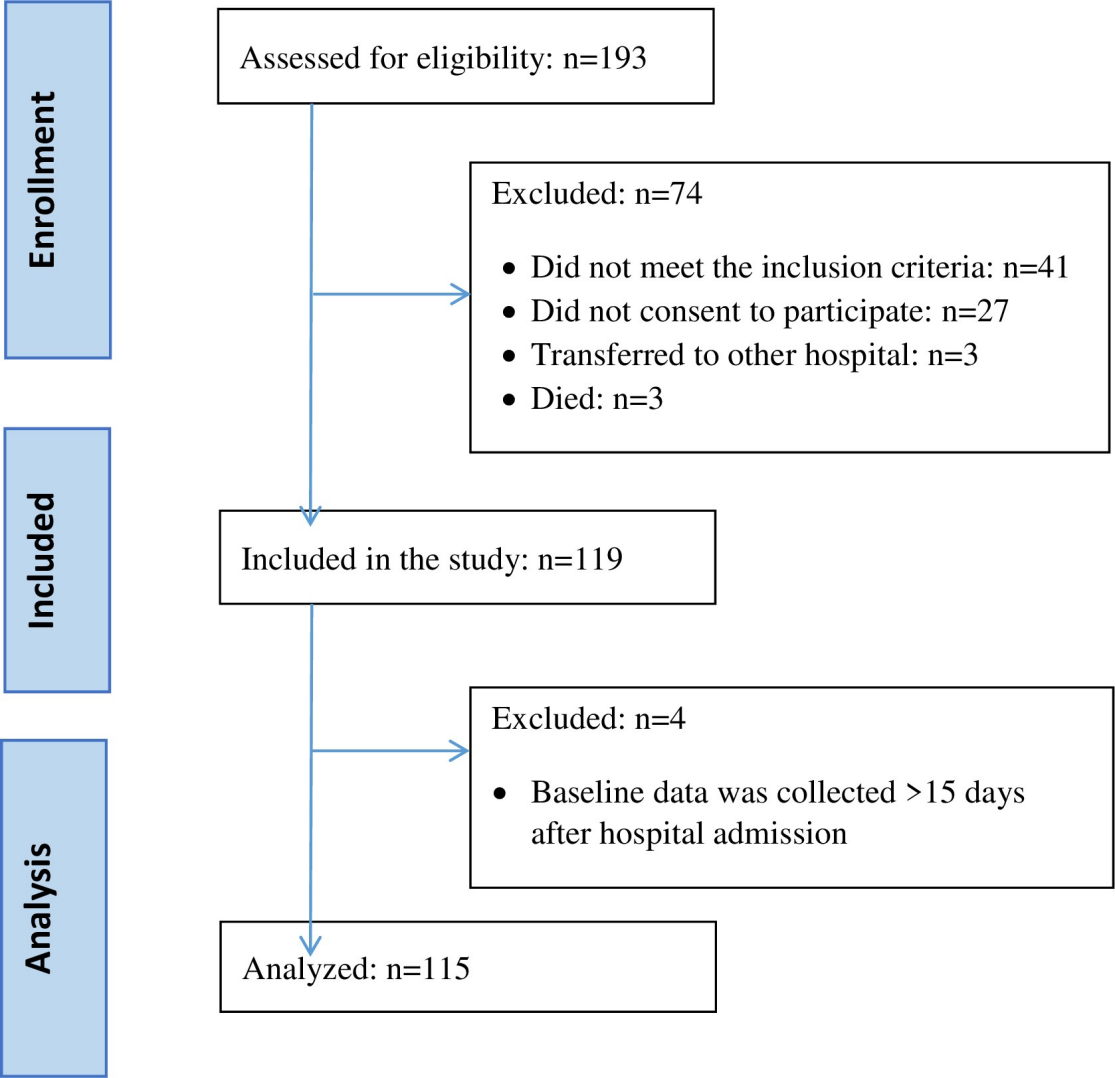
Flow chart showing the recruitment and inclusion of patients in the study.

The Post-Stroke Fatigue Study is a longitudinal, observational study that recruited stroke patients admitted to either of two hospitals in Oslo and Buskerud counties between March 2007 and September 2008. The main inclusion criterion was first-ever stroke according to the International Classification of Disease (ICD-10 diagnoses I60-I64). Patients also had sufficient cognitive function to consent to participate, be fully conscious, and be oriented to person, place, and time. If unable to point to response alternatives on a questionnaire, they were excluded. Of the 193 patients with a diagnosis of first-ever stroke admitted to these two hospitals, 119 were included in the study. Four were subsequently excluded because baseline data was collected more than 15 days after hospital admission. Thus, the sample for analyses includes 115 patients. Details of the recruitment and inclusion process are described in Fig 1.

Data were collected at four time points (T1-T4): during the acute phase (within 14 days of the stroke), and at 6, 12 and 18 months after the stroke. The data were collected through structured interviews and by use of standardized questionnaires. Data on stroke type, lesion location and comorbid conditions were collected from the patients’ medical records.

### Measurements

#### Sociodemographic variables

Data on sex, age, use of medications, and time since hospital admission were collected from the patients’ medical records. Data on cohabitation status (i.e., living alone or with others), level of education, employment status and physical functioning were collected through a structured interview.

#### Medical characteristics

Stroke type and lesion location: Computed tomography (CT) scans were taken of all patients upon admittance to hospital. Based on the radiologists’ descriptions, the stroke was categorized into one of the following three types: ischemic infarct, hemorrhage and negative findings/clinical stroke. The location of the lesion was categorized as left, right or bilateral. Some lesions from previous undiagnosed strokes showed up on CT scans and were included in the classification of stroke type and location.

Comorbidities included present or past conditions. This analysis focused on conditions considered relevant to fatigue, specifically those affecting circulatory, respiratory, endocrine, nervous, mental, or muscular systems.

Body mass index (BMI) was calculated as the patient’s weight in kilograms divided by the square of their height in meters. Weights and heights were obtained from the patients’ medical records.

#### Symptom-related measures

Fatigue was measured with the Fatigue Severity Scale (FSS). The FSS is a 9-item instrument where higher mean scores indicate higher degrees of fatigue [15]. The FSS has been shown to be both valid and reliable in different clinical groups [16]. In a study by Nadarajah et al., the FSS had a Cronbach’s alpha of >0.90 in samples of stroke patients and healthy controls. The FSS was also strongly correlated with visual analog scales of fatigue (r = 0.60) [16].

Pre-stroke fatigue was assessed through a clinical semi-structured interview. Patients were asked whether they had experienced substantial fatigue, defined as affecting their ability to perform daily activities, before the stroke, and if they had, whether this had lasted less than 3 months, 3–6 months or for more than 6 months. Patients who reported substantial fatigue lasting at least 3 months before the stroke were defined as having pre-stroke fatigue [6].

Depression was measured with the Beck Depression Inventory (BDI), a 21-item self-report questionnaire. BDI scores of 0–9 indicate normal mood, 10–14 mild depression, 15–24 moderate depression and 25 and above severe depression [17] Studies on psychometric properties of the inventory has showed good internal consistency, concurrent validity and a good ability to discriminate subtypes of depression [18]. In this sample the BDI-items had a Cronbach’s alpha of 0.855.

Sleep quality was assessed with the Pittsburgh Sleep Quality Index (PSQI), a self-report questionnaire consisting of 19 items that assess sleep quality over a one-month time period. A PSQI sum score is calculated and ranges from 0 to 21, where higher scores indicate worse sleep quality. A score higher than 5 is indicative of sleep disturbance. An assessment of the index’ psychometric properties showed acceptable internal homogeneity, reliability and validity [19].

Activities of Daily Living (ADL) with regard to the patient’s level of dependency- independency were assessed with the Barthel Index (BI) for ADL, a scale of 10 items where higher scores represent higher levels of independence [20]. The BI score was based on the patients’ self-rating on the questionnaire or patient interviews. The instrument has been shown to be a reliable and valid measure of basic ADL function in patients with stroke. As we did not have a direct measure of the severity of stroke, such as the National Institute of Health Stroke Scale (NIHSS), a tool used by healthcare providers to objectively quantify the impairment caused by stroke, the BI score served as a measure of severity of function loss following stroke.

### Data analysis

Statistical analyses were performed using SPSS software version 23 (IBM Corp, Armonk, NY, USA) and Stata software version 13 (Stata Corp, College Station, TX, USA).

Growth mixture modelling (GMM) in the GLLAMM package in Stata [21] was used to identify latent classes, i.e. subgroups of patients based on their FSS scores at the four time points. GMM allows for the estimation of more than one growth curve, for previously unidentified subgroups that change differently over time. GMM has benefits over alternative methods, as it can allow for variations between individuals and subgroups, not only between individuals and population averages. GMM employs a model-based approach to calculate the probability of membership in each class, and also quantifies uncertainty in class membership [22]. The number of latent classes identified was based on likelihood values and stability of the estimated classes. In general, mixture models aim to uncover unobserved heterogeneity in a population and to find substantively meaningful groups of people that are similar in their responses to measured variables or trajectories [23].

SPSS was used to calculate descriptive statistics and perform hypothesis testing about group differences and associations between variables. For hypothesis testing, the significance level was set at 0.05.Univariate associations between pre-stroke fatigue and comorbidities were evaluated using Chi-square tests, or Fishers Exact test when expected cell frequencies were <5. Associations between sociodemographic, medical, and symptom-related characteristics and the latent classes (i.e., fatigue trajectories) were evaluated by using ordinal regression for both univariate and multivariable analyses. Variables included in the multivariable analyses were based on theoretical importance (i.e., gender) or a p-value of ≤ 0.10 in the univariate analyses. Effect sizes for the associations are reported as odds ratios (ORs) with 95% confidence intervals (CI).

### Ethics

This study was approved by the Regional Medical Research Ethics Committee of Health for the South East of Norway (Ref #2.2007.90) and by the Data Protection Officer at Oslo University Hospital. All patients provided written informed consent prior to participation.

## Results

One hundred and fifteen patients were included in the analyses (see Fig 1 for details). Most participants were male (n = 68, 59%), lived with a partner or at least one other person (n = 72, 63%) and the mean age was 68.3 (SD 13.3) years. Sociodemographic, medical and symptom-related characteristics of the sample are shown in Table 1. Because the vast majority of patients (77%) had at least one comorbidity relevant to fatigue and only four patients reported more than three, subsequent analyses compared patients with at least two comorbidities to those with less than two.

**Table 1:** Demographic, clinical, and stroke-related characteristics of the sample (N = 115).

Characteristic	Total sample (N = 115)	Class 1	Class 2	Class 3
		Low fatigue (n = 23)	Moderate fatigue (n = 52)	High fatigue (n = 40)
***Sociodemographics***				
**Sex**, n (%)				
Male	68 (59%)	14 (60%)	34 (61%)	20 (50%)
Female	47 (41%)	9 (40%)	18 (39%)	20 (50%)
**Age**, mean (SD)	68.3 (13.3)	67.1 (9.2)	67.3 (14.2)	70.3 (14.1)
**Cohabitation**, n (%)				
Living with others	72 (63%)	18 (78%)	32 (62%)	22 (55%)
Living alone	43 (37%)	5 (22%)	20 (38%)	18 (45%)
**Education**, n (%)				
Low (<high school)	83 (70%)	15 (65%)	37 (67%)	31 (78%)
High (≥high school)	32 (30%)	8 (35%)	15 (33%)	9 (22%)
**Employment**, n (%)				
Working at T1^a^	28 (24%)	8 (35%)	14 (27%)	6 (15%)
Working at T4^b^	15 (13%)	4 (17%)	8 (15%)	3 (8%)
***Medical variables***				
Stroke type				
schemic	90 (78%)	19 (83%)	38 (73%)	33 (82%)
Haemorrhage	7 (6%)	1 (4%)	3 (6%)	3 (8%)
Negative findings/clinical stroke	18 (16%)	3 (13%)	11 (21%)	4 (10%)
Side of stroke lesion				
Right	31 (27%)	10 (43%)	12 (23%)	9 (22%)
Left	29 (25%)	2 (9%)	16 (31%)	11 (28%)
Bilateral	20 (17%)	5 (22%)	7 (13%)	8 (20%)
Unknown	35 (31%)	6 (26%)	17 (33%)|	12 (30%)
Comorbidities ^c^ n (%)				
None	27 (23%)	8 (35%)	14 (27%)	5 (12%)
1	49 (43%)	11 (48%)	26 (50%)	12 (30%)
2 or more	39 (34%)	4 (17%)	12 (23%)	23 (58%)
BMI, mean (SD)	26.2 (5.1)	25.6 (5.5)	26.0 (4.7)	26.7 (5.5)
***Symptom-related variables***				
Pre-stroke fatigue, n (%)	34 (30%)	1 (4%)	13 (25%)	20 (50%)
BDI, mean (SD)	9.6 (7.6)	5.0 (5.6)	9.0 (6.9)	13.0 (8.1)
PSQI, mean (SD)	6.9 (3.6)	5.2 (3.6)	6.9 (3.6)	7.9 (3.3)
Barthel Index, mean (SD)	17.7 (4.1)	18.0 (5.0)	18.2 (3.5)	16.9 (4.1)

Abbreviations: Barthel Index = measure of independence with activities of daily living; BDI = Beck Depression Inventory II; BMI = body mass index; PSQI = Pittsburgh Sleep Quality Index.

^a^ T: At the time of the stroke

^b^ T4: Within 18 months after the stroke

^c^ Comorbidities relevant to fatigue included chronic conditions affecting the circulatory, respiratory, endocrine, nervous, mental, or muscular systems.

**Fig 2:**
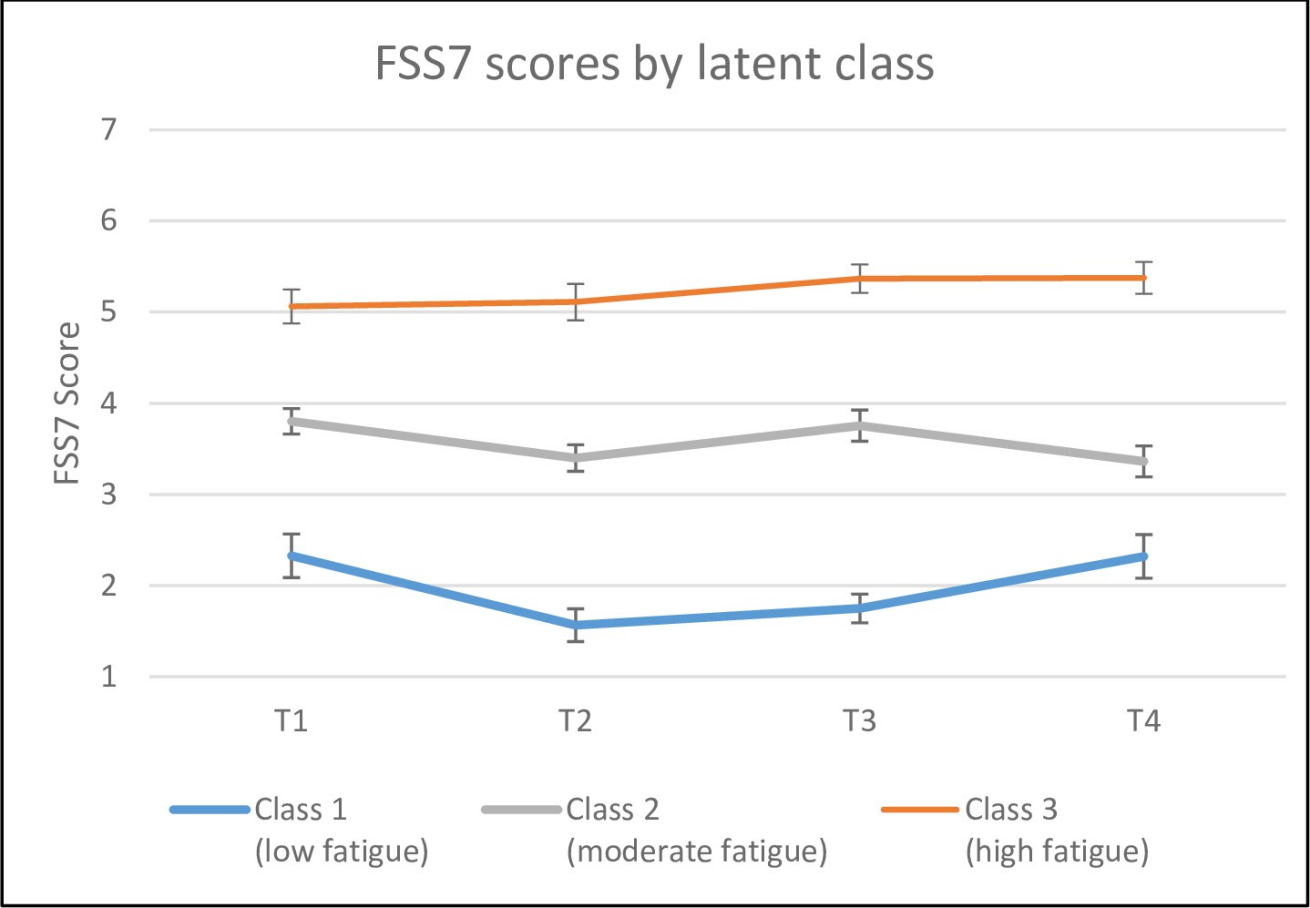
FSS7 scores by latent class.

Using GMM, three latent classes of fatigue trajectories over 18 months were identified. As shown in Fig 2 and Table 2, the mean FSS scores stayed relatively stable across all four time points in all three groups, but each group differed in the degree of fatigue reported across time. Therefore, the three classes were labeled low (n = 23, 20%), moderate (n = 52, 45%) and high fatigue (n = 40, 35%).

**Table 2:** Mean (SD) FSS scores at each time point, overall and by fatigue class.

Time	Total sample (N = 115)	Class 1	Class 2	Class 3
		Low fatigue (n = 23)	Moderate fatigue (n = 52)	High fatigue (n = 40)
T1: acute phase	3.95 (1.47)	2.33 (1.15)	3.80 (1.01)	5.06 (1.18)
T2: 6 months	3.62 (1.63)	1.56 (0.80)	3.40 (1.01)	5.11 (1.18)
T3: 12 months	3.86 (1.64)	1.74 (0.72)	3.75 (1.17)	5.37 (0.89)
T4: 18 months	3.76 (1.58)	2.32 (1.10)	3.36 (1.15)	5.38 (0.96)

While the sample’s mean depression level (mean BDI score 9.6, SD 7.6) during the acute phase was below the cutoff score of 14, the BDI scores of the high (mean 13.0, SD 8.1) and moderate (mean 9.0, SD 6.9) fatigue groups were significantly higher (p < .001 and p = .027, respectively) than in the low group (mean 5.0, SD 5.6). Whereas 13% of those in the low fatigue group suffered from mild to severe depression based on BDI scores, 49% suffered from mild to severe depression in the high fatigue group.

In the univariate ordinal regression analyses, the baseline patient characteristics that were significantly associated with having a higher fatigue trajectory were being over 75 years of age and having pre-stroke fatigue, having at least two comorbidities relevant to fatigue, mild-severe depression, disturbed sleep, or at least some ADL impairment (see Table 3). Living alone and not working at T1 and T4, were not significantly related to a patient’s fatigue trajectory, but because their p-values were <0.10, these variables were included in the multivariable analysis. The patient’s gender, education, stroke type and location, and BMI were unrelated to their fatigue trajectory, but patient gender was included in the multivariable model based on prior findings of gender differences in fatigue.

**Table 3:** Univariate and multivariable ordinal regression analyses of fatigue class.

Independent variables	Fatigue class, n (%)			Ordinal regression	
	Low (n = 23)	Medium (n = 52)	High (n = 40)	Univariate OR (CI)	Multivariable OR (CI)
***Sociodemographics***					
**Sex**					
Female	9 (39%)	18 (35%)	20 (50%)	1.50 (0.75, 3.03)	1.13 (0.50, 2.56)
Male	14 (61%)	34 (65%)	20 (50%)	reference	reference
**Age**					
<60 years	3 (13%)	12 (23%)	8 (20%)	1.72 (0.68, 4.34)	3.17 (0.87, 11.46)
60–75 years	14 (61%)	24 (46%)	14 (35%)	reference	reference
>75 years	6 (26%)	16 (31%)	18 (45%)	**2.23 (1.02, 4.90)**	1.90 (0.60, 3.74)
**Cohabitation**					
Living with others	18 (78%)	32 (62%)	22 (55%)	reference	reference
Living alone	5 (22%)	20 (38%)	18 (45%)	**1.86 (0.91, 3.82)**	1.79 (0.71, 4,53)
**Education**					NI
High	8 (35%)	15 (29%)	9 (22%)	reference	
Low	15 (65%)	37 (71%)	31 (78%)	1.52 (0.71, 3.26)	
**Employment at T1**					
Working	8 (35%)	14 (27%)	6 (15%)	reference	reference
Not working	15 (65%)	38 (73%)	34 (85%)	**2.13 (0.95, 4.77)**	**4,61 (1,36,15,70)**
***Medical variables***					
**Stroke type**					NI
Infarct	19 (83%)	38 (73%)	33 (82%)	1.32 (0.51, 3.40)	
Hemorrhage	1 (4%)	3 (6%)	3 (8%)	1.84 (0.35, 9.54)	
No signs	3 (13%)	11 (21%)	4 (10%)	reference	
**Stroke side** (n = 80)					NI
Left	2 (12%)	16 (46%)	11 (39%)	0.61 (0.21, 1.75)	
Right	10 (59%)	12 (34%)	9 (32%)	1.38 (0.47, 4.01)	
Bilateral	5 (29%)	7 (20%)	8 (29%)	reference	
**Comorbidities**^**a**^					
No comorbidities	7 (30%)	16 (31%)	5 (12%)	reference	reference
Comorbidities	16 (70%)	36 (69%)	35 (88%)	**4,42 (2,024.78)**	4,52(1,8511,10)
**BMI** (n = 108)					NI
≤25	11 (48%)	19 (39%)	15 (42%)	reference	
>25	12 (52%)	30 (61%)	21 (58%)	0.88 (0.43, 1.80)	
***Symptom-related variables***					
**Pre-stroke fatigue**					
Yes	1 (4%)	13 (25%)	20 (50%)	**4.97 (2.18, 11.3)**	**4,92 (1,84,13,20)**
No	22 (96%)	39 (75%)	20 (50%)	reference	reference
**Depression** (n = 112)					
None, BDI 0–13	20 (87%)	39 (78%)	20 (51%)	reference	reference
Mild-severe, BDI ≥14	3 (13%)	11 (22%)	19 (49%)	**3.87 (1.71, 8.73)**	2.20 (0.85, 5.75)
**Sleep Quality** (n = 113)					
Normal, PSQI = <5	11 (48%)	16 (32%)	6 (15%)	reference	reference
Disturbed, PSQI>5	12 (52%)	34 (68%)	34 85%)	**3.06 (1.39, 6.73)**	2.41 (0.97, 5.98)
**ADL Independence** (n = 114)					
Impaired (BI<20)	4 (17%)	19 (36%)	20 (51%)	**2.66 (1.27, 5.54)**	1,99 (0854,65
Not impaired (BI = 20)	19 (83%)	33 (64%)	19 (49%)	reference	reference

Bold odds ratios and CIs have p<0.10 for univariate analyses and p<0.05 for multivariable analyses.

Abbreviations: ADL = activity of daily living; BDI = Beck Depression Inventory II; BI = Barthel Index; BMI = body mass index; CI = 95% confidence interval; NI = not included in the multivariable model because the variable was not associated (p<0.10) with fatigue class in univariate analysis; OR = odds ratio; PSQI = Pittsburgh Sleep Quality Index.

^a^ Comorbidities relevant to fatigue included chronic conditions affecting the circulatory, respiratory, endocrine, nervous, mental, or muscular systems.

In the multivariable ordinal regression analysis, the only baseline patient characteristics associated with having a higher fatigue trajectory were not working, having multiple comorbidities and having pre-stroke fatigue (see Table 3). Although neither depression nor sleep disturbance were associated with fatigue trajectory when controlling for other relevant factors, when either one of these factors was omitted from the model, the other became significant (depression OR = 2.69, 95% CI 1.06, 6.88; sleep OR = 283, 95% CI 1.17, 6.84), suggesting that their associations in the multivariable model were affected by multicollinearity. Nonetheless, having pre-stroke fatigue was the strongest predictor of a worse fatigue trajectory, with patients reporting persistent fatigue (>6 months) prior to their stroke having nearly 5 times higher odds of a higher post-stroke fatigue trajectory than patients without pre-stroke fatigue (OR = 5.92, 95% CI1,84, 15.5). A univariate analyses showed no significant relationships between pre-stroke fatigue and pre-stroke comorbidities in regards to specific conditions which might be associated with fatigue, such as those affecting the circulatory, endocrine, neurological or muscular systems. Pre-stroke fatigue was not only associated with fatigue class, but was also associated with having multiple pre-existing comorbidities (p = .0180), particularly a mental health comorbidity (p = .002). However, pre-stroke fatigue was not specifically associated with comorbid conditions affecting the circulatory, respiratory, endocrine, neurological or muscular systems. Of the 34 patients reporting pre-stroke fatigue, most (59%) had a high fatigue trajectory in the year after stroke, whereas only one of these patients (3%) had a low fatigue trajectory. In contrast, of the 81 patients without pre-stroke fatigue, only 25% had a high fatigue trajectory, and a similar proportion (27%) had a low fatigue trajectory.

## Discussion

To our knowledge, this is the first study that has explored a sample of first-ever stroke patients’ trajectories of fatigue from the acute phase through the first 18 months post-stroke. Firstly, in this sample, the GMM analyses identified three classes with distinct trajectories of fatigue during the first 18 months post stroke. Our study adds to the current body of knowledge by indicating that it might be possible to identify many of those at risk for PSF at an early stage following a first-ever stroke.

A notable finding was that the severity of fatigue symptoms within each of the three classes was quite stable across the four time points. Thus, those who were fatigued at an early stage generally remained fatigued 18 months later, and those who experienced a low degree of fatigue at an early stage continued to experience a low degree of fatigue 18 months after stroke. Our findings thus suggest that distinct trajectories of fatigue intensity following stroke were present in the sample and that the potential onset of the symptom occurs as early as the acute phase. A common belief about fatigue in the acute phase after stroke is that it is a normal consequence of acute injury and will resolve as the patient recuperates. However, as none of the trajectories identified in this study reflect this pattern of fatigue resolution, our findings do not provide empirical evidence to support this belief.

The multivariable analyses identified pre-stroke fatigue as significantly associated with post-stroke fatigue trajectory. Those in the high fatigue class were the most likely to report pre-stroke fatigue, which is consistent with previous literature [6]. There might be factors that underlie both pre- and post-stroke fatigue, such as pre-existing cardiovascular disease and other chronic medical conditions, poor health, lifestyle factors, vulnerability to stress or mental health conditions, such as anxiety and/or depression.

In the univariate analyses, we found an association between higher fatigue trajectories and depressive symptoms and sleep quality, respectively. In the multivariable analyses, however, we did not find a significant association between either of these variables and fatigue class. Regarding depression and fatigue, there may be an overlap in symptoms that makes a clear distinction between these two diagnoses difficult using standardized measures. It might be that some of the symptoms that correlate with fatigue most strongly are also those that overlap the most, such as sleep disturbance, lack of energy and lack of initiative. In line with this, patients with the high fatigue trajectory reported significantly worse sleep quality than those with lower fatigue trajectories, although the mean PSQI score was within the clinical range for all three fatigue classes. This finding is also in line with previous research [24]. PSF and post-stroke depression also share common risk factors, such as premorbid psychiatric illness, social isolation and functional impairments [25]. Even though fatigue and depression might be related, fatigue can also occur without depression [26]. In our study, 51% of the patients in the high fatigue class had BDI scores in the “no depression” range.

There might be clusters of symptoms that are associated with fatigue, including depression and sleep disturbance. These symptoms, along with fatigue, might be part of a self-perpetuating cycle. Such interactions need to be explored in further research.

Although there is no data on cognitive function in this sample, the symptom-related characteristics of the three classes indicate that more symptom burden, such as more depressive symptoms, more severe sleep disturbance, more dependency in ADL and a higher degree of comorbidity, is related to a higher degree of fatigue over time, although the possible interactions between these factors or possible common predisposing factor(s) remain unclear. Thus, it could be that there are subgroups within the classes as well. For instance, there may be distinct subgroups within the high fatigue class, both in relation to causes of fatigue and trajectories. It might be that some stroke patients suffer from fatigue due to physical impairments, such as hemiparesis, reduced balance or gait, or visual disturbances, while others might suffer from fatigue due to psychological stress, life stressors or inadequate coping styles. In others, cognitive impairments, such as reduced attention or vigilance, might be the driving force for fatigue.

The large variability in estimates of PSF’s prevalence across studies reflects differences in definitions and inclusion criteria, assessment at different time points after stroke onset, and use of different fatigue measures and different cut-offs on fatigue scales. This variability also makes comparison between studies challenging [24]. For instance, the 9-item Fatigue Severity Scale (FSS) is the most widely used instrument to measure PSF, but some studies have used a cut-off score of 4 to define fatigue [27, 28], while others have categorized FSS scores into three groups; <4 indicates no/mild fatigue, 4–4.9 indicates moderate fatigue, and > = 5 indicates severe fatigue [6]. It has been argued that using a cut-off of 4 leads to an overestimation of caseness, as 46.7% of a sample of 1893 randomly-selected Norwegians scored in this range. Therefore, using a cut-off of 5 to identify severe fatigue has been proposed [29]. Our findings support a cut-off of 5 to identify severe fatigue cases, given that the high fatigue group had mean FSS scores above 5 at all time points. In comparison, the moderate fatigue group on average scored between 3 and 4.

In our sample, 35% of patients were in the high fatigue class, exceeding the number of those in the general population with FSS scores within the range for fatigue (14–23%). Nevertheless, fatigue is common in many different diagnoses [28], as well as in the population in general [29], so it might be that fatigue is a generic symptom in many medical chronic conditions. If fatigue severity can be explained by transdiagnostic factors, then patients with different diagnoses may be able to benefit from the same interventions [30].

### Study strengths and limitations

This is, to our knowledge, the first study identifying distinct classes of fatigue trajectories, including a measure of fatigue in the acute phase and using a statistical approach that allowed us to identify subgroups with distinct trajectories of fatigue. The longitudinal design with four time points over 18 months is another strength of this study, as it allows for identification of trajectories over time. The advantages of GMM compared to more traditional statistical analyses are that it can identify multiple unobserved sub-populations and allows for differences between the identified groups to be examined. Using the classes high, moderate and low fatigue circumvents the challenge of which cut-off score to use for defining PSF cases, as described above.

This study also has limitations. First, the results may be influenced by selection/inclusion bias. We excluded patients unable to respond to the questionnaire. This group may have a significant prevalence of disability-related fatigue. Among those included, there was a tendency for those in the high fatigue group to be older and more dependent in ADL. As a whole, however, the sample is characterized by being fairly independent in ADL. As we have no information on clinical characteristics of eligible patients who did not consent to participate, we do not know what their ADL function was or whether the final sample is representative of the larger population from which it was drawn. Also, it is a limitation that we do not have data from a direct measure of stroke severity, such as the NIHSS score. Furthermore, limiting the inclusion to first-ever stroke patients makes the sample less representative of the stroke population as a whole. Previous strokes are prevalent and may render patients more vulnerable to fatigue, as well as to a higher degree of other symptoms and/or medical conditions.

Moreover, the sample may have been too small to find significant differences in fatigue across stroke diagnoses, and between those who have a positive finding on MR/CT and those who have clinical strokes. It remains uncertain whether the presence of a lesion or lesion site is of importance in the development of chronic fatigue after stroke. While BMIs were higher in the high fatigue class, the study might have been underpowered to detect statistically significant differences between the three classes on this measure.

The measurement of pre-stroke fatigue was based on retrospective self-report, which renders the measure vulnerable to recall bias, and we have no information on its psychometric properties. Furthermore, the measurement of pre-stroke fatigue was not based on an FSS score, as was the measurement of post-stroke fatigue, and thus, we cannot determine whether the degree of fatigue changed, such that those who were already fatigued became even more fatigued, or whether the degree of fatigue remained stable after stroke.

As PSF is a complex phenomenon, future research on post-stroke fatigue trajectories should include a wide variety of measures in addition to those included in this study, such as objective measures of physical function, pain, cognitive function and life stressors. In addition, psychological factors should be explored, such as coping skills, self-efficacy and personality traits as mediating factors for both the perception and implications of fatigue. Some of these factors will be included in a currently ongoing study that aims to identify predictors and subgroups of post-stroke fatigue.

To address the complexity a larger study with a larger sample in the high fatigue class is needed in order to have sufficient heterogeneity in regards to age, physical, cognitive and psychological factors to find possible clusters. If fatigue severity can be explained by generic factors, interventions aimed at ameliorating fatigue do not have to be diagnosis-specific.

## Conclusion

This study identified three distinct classes of trajectories of fatigue following stroke, with stable FSS scores from the acute phase through 18 months post-stroke. Furthermore, the study identified pre-stroke fatigue, multiple comorbidity, and not working as predictors of a higher fatigue class. Fatigue in the acute phase also seems to be predictive of long-lasting fatigue. This knowledge may be useful for identifying patients at risk of developing chronic fatigue, and for developing individually tailored treatment programs for stroke patients. If fatigue severity can be explained by transdiagnostic factors, it might also be possible for patients with different diagnoses to be included in similar intervention programs.

Further research on PSF in a representative sample is needed to verify our findings and to clarify the role of depression, sleep quality and other factors such as cognitive function, life stressors, and both psychological and physical functioning. The implication of potentially different PSF subgroups with different underlying causes would be that assessment should be broad and that interventions should be tailored to the patient as much as possible.
